# Proteins and Bioactive Peptides in High Protein Content Foods

**DOI:** 10.3390/foods10061186

**Published:** 2021-05-25

**Authors:** Fidel Toldrá, Leticia Mora

**Affiliations:** Instituto de Agroquímica y Tecnología de Alimentos (CSIC), Avenue Agustín Escardino 7, Paterna, 46980 Valencia, Spain; ftoldra@iata.csic.es

Foods and their industry by-products constitute very good sources of bioactive peptides, which can be naturally generated during processing but are also extensively produced through enzymatic hydrolysis, microbial fermentation, and even during gastrointestinal digestion in the human body. These sequences of bioactive peptides remain inactive while forming part of the parent protein but once released, they exert bioactivity. The generated peptides are frequently transported through the blood stream to different target organs, where they exert their biological function.

The use of commercial proteolytic enzymes for the generation of bioactive peptides is the most extensively used methodology to obtain an optimized production yield. These enzymes are obtained from animal, plant, or microbial sources, and frequently endo- and exopeptidase activities are combined in order to obtain the best results, as shown in this Special Issue. In this regard, the manuscript produced by Fan et al. reported a spent hen protein that was hydrolyzed using the enzymes Thermoase PC10F, Protease M, Alcalase 2.4 L, Protex 50 FP, Protex 26 L, Protex 6 L, pepsin, trypsin, and their combination. The hydrolysates prepared with Protex 26 L, pepsin, and Thermoase showed the best angiotensin-converting enzyme I (ACEI)-inhibitory activity and peptide yield. The same hydrolysates were assayed for their best angiotensin-converting enzyme 2 (ACE2) upregulating, antioxidant, and anti-inflammatory activities and the effect of a simulated gastrointestinal digestion was also evaluated. However, it is very common that in vitro activities do not correlate with in vivo activities and, in this study, only the Thermoase hydrolysate reduced blood pressure significantly when given orally to spontaneously hypertensive rats at a daily dose of 1000 mg/kg body weight [1]. 

Regarding commercial enzymes, Alcalase has been extensively used in the generation of bioactive peptides from different food matrices, mainly due to the strong endopeptidase activity of this enzyme. In this regard, the article by Hayes and Mora in this Special Issue describes the potential heart health benefits of Alcalase snail hydrolysates after the in vitro ACEI-inhibitory activity measurement and the identification of main peptides using mass spectrometry in tandem [2]. Mazloomi et al. assayed the biological activity of an Alcalase hydrolysate of orange seed by-products that proved to exert antioxidant, ACEI-inhibitory, and α-amylase and β-glucosidase inhibitory activities, and similar bioactivities remained after a simulated gastrointestinal digestion. These studies confirm the high potential of the Alcalase enzyme for the generation of bioactive peptides that could be later used as nutritional supplements or functional enhancers in functional foods [3].

Bioactive peptides can also be released during the gastrointestinal digestion of ingested foods. However, the generated bioactive peptides must remain intact through gastrointestinal digestion and must be intact when they cross the intestinal barrier and reach the blood stream to exert their physiological action. Frequently, gastrointestinal peptidases, such as pepsin, trypsin, and chymotrypsin, participate in the digestion of food proteins and oligopeptides, generating novel fragments that also have the potential to act as a bioactive.

In this sense, the peptides generated after a simulated gastrointestinal digestion of cocoa seed proteins have been studied by Coronado-Cáceres et al. to determine the pancreatic lipase inhibitory effect using in silico and in vitro approaches. The predicted properties of the peptides, including a good probability of absorption and a low probability of hepatotoxicity, being non-carcinogenic, and non-mutagenic, were confirmed using an in vivo high-fat diet obese rat model, proving that cocoa protein has anti-obesity potential by inhibiting pancreatic lipase [4]. In another study by Cerrato et al. that is included in this Special Issue, different peptide fragments were obtained from yellowfin tuna muscle using a simulated gastrointestinal digestion, and their antimicrobial activity towards Gram-positive and Gram-negative bacteria was investigated using two different proteomic approaches for the identification of peptides according to their molecular mass [5].

Very frequently, the processing of food includes the use of treatments that could affect the peptides themselves or their bioactivity, making it necessary to study the stability of bioactive peptides under typical thermal-processing conditions. In this sense, the effect of different cooking methodologies on antioxidant activity after the in vitro gastrointestinal digestion of different legume pastes has been evaluated by Gallego et al. in this Special Issue [6]. Cooking followed by gastrointestinal digestion improved the protein digestibility and antioxidant activity of the legumes, which was attributed to the released peptides and amino acids more than the free phenolics.

The experimental design can be simplified by using bioinformatics for computer simulation in most of the steps. In this respect, different empirical and in silico approaches have also been used by Iwaniak et al., as reported in this Special Issue, to identify the bioactive peptides naturally generated by the collagen protein of different animal species using quantitative parameters [7]. The BIOPEP-UWM-implemented quantitative criteria includes the frequency of the release of fragments with a given activity by selected enzyme(s) (AE), the relative frequency of the release of fragments with a given activity by selected enzyme(s) (W), and the theoretical degree of hydrolysis (DH). To find out whether a protein can release peptides and which enzyme has an adequate potential to produce them, the analysis of the proteins for which AE and W had relatively comparable values is recommended. Based on this, it was observed that pepsin and/or trypsin were effective producers of ACEI and/or dipeptidyl peptidase IV (DPP-IV) inhibitors during collagen hydrolysis [7].

Despite this, the identification and quantitation of bioactive peptides using empirical approaches is frequently necessary and peptidomic methodologies based on mass spectrometry in tandem are the most used. In this Special Issue, a label free quantitative untargeted and targeted approach based on liquid chromatography coupled to mass spectrometry in tandem (LC−MS/MS) has been used by Aiello et al. to analyze the protein profile of different lupin-enriched pasta samples, using tryptic and peptic digestions [8]. The untargeted methodology permits a comparison between the profiles of proteins, whereas the targeted method, based on Multiple Reaction Monitoring, permits the absolute quantification of the main components of lupin.
